# Hydrocortisone versus dexamethasone in cerebral salt-wasting after aneurysmal subarachnoid hemorrhage

**DOI:** 10.1016/j.bas.2026.105967

**Published:** 2026-02-12

**Authors:** Leander Steger, Benoit Liquet, Kevin Agyemang, Moritz Freistühler, Antonio Di Ieva, Walter Stummer, Christian Ertmer, Eric Suero Molina

**Affiliations:** a Department of Neurosurgery, University Hospital Münster, Muenster, Germany; b School of Mathematical and Physical Sciences, Macquarie University, Sydney, Australia; c Laboratoire de Mathématiques et de ses Applications, E2S-UPPA, Université de Pau & Pays de L'Adour, France; d Macquarie Medical School, Faculty of Medicine, Health and Human Sciences, Macquarie University, Sydney, New South Wales, Australia; e Controlling Department, University Hospital Munster, Muenster, Germany; f Department of Anesthesiology, Intensive Care and Pain Medicine, University Hospital Münster, Muenster, Germany

**Keywords:** Hyponatremia, Mineralocorticoids, Cerebral salt wasting, Non-traumatic subarachnoid hemorrhage

## Abstract

**Introduction:**

Cerebral salt wasting syndrome (CSW) is frequently observed in aneurysmal subarachnoid hemorrhage (SAH) patients and results in excessive natriuresis with decreased extracellular fluids, leading to hyponatremia and hypovolemia. Hyponatremia is associated with an increased complication rate and potential mortality.

This study compares hydrocortisone and dexamethasone for CSW-associated hyponatremia prophylaxis after non-traumatic SAH.

**Methods:**

This retrospective cohort study analyzed data from 510 consecutive patients with non-traumatic SAH who were admitted to the University Hospital of Münster, Germany, between October 2009 and December 2019. Hyponatremia was defined as blood sodium levels <130 mmol/L. We compared 188 patients treated with dexamethasone and 322 with hydrocortisone, focusing on the incidence of hyponatremia (<130 mmol/L) and CSW, defined as sodium levels <135 mmol/L with a negative fluid balance.

**Results:**

Hyponatremia (Na(p) < 130 mmol/L) developed in 87 patients (25.0% dexamethasone, 12.4% hydrocortisone; *p* = 0.0004). Median treatment durations were 9.0 days for dexamethasone (IQR, 5.0-15.0 days) and 10.0 days for hydrocortisone (IQR, 8.0-12.8 days). Average daily doses were 9.2 mg (±4.3) of dexamethasone and 114.3 mg (±81.9) of hydrocortisone. Simultaneous negative fluid balance and hyponatremia (Na(p) < 135 mmol/L) occurred in 203 patients (39.8%) (47.3% dexamethasone vs. 35.4% hydrocortisone) *(p=*0.0079, OR: 1.64, 95% CI: 1.14-2.37). For hyponatremia alone (Na(p) < 130 mmol/L), multivariable analysis showed an OR of 0.21 *(p=0.00021*, 95%CI: 0.09-0.48) between both groups, indicating a 4.8 times higher risk in the dexamethasone group.

**Conclusion:**

Our findings indicate that hydrocortisone is associated with a lower frequency of CSW-associated hyponatremia following non-traumatic SAH as compared to dexamethasone.

## Highlights

• Retrospective cohort of 510 non-traumatic SAH patients comparing hydrocortisone vs dexamethasone for CSW-related hyponatremia.
• Severe hyponatremia (<130 mmol/L) occurred less often with hydrocortisone (12.4%) than deamethasone (25%), p ≈ 0.0002.
• CSW proxy (Na<135 mmol/L + negative daily fluid balance) occurred less often with hydrocortisone(35.4% vs 47.3%, p = 0.008).
• Hydrocortisone showed fewer recurrences: 95% remained hypoantremia-free by day 7 vs 79% with dexamethasone (p<0.0001).
• Risk factors: lower sodium (p = 0.012) and older age (p = 0.034) raised risk; higher steroid dose trended protective (p = 0.058).

## 1 Introduction

Cerebral salt wasting (CSW) affects patients with brain diseases, such as subarachnoid hemorrhage (SAH) or central nervous system (CNS) infections (Cerdà-Esteve et al., 2008). It causes inadequate renal sodium loss, leading to hyponatremia and hypovolemia due to extracellular fluid loss.

CSW-associated hyponatremia is commonly seen in patients with aneurysmal subarachnoid hemorrhage (aSAH) and CNS diseases, including stroke, infection, or after brain surgery (Yee et al., 2010; Kaiya et al., 2019; Momi et al., 2010).

A recent systematic review and meta-analysis reported a pooled incidence of hyponatremia in 37% patients with aSAH during hospital admission (Gillespie et al., 2025). Therefore, prevention of hyponatremia after SAH is critical because the global aneurysmal SAH incidence is ~6.1 per 100,000 person/year (Hoh et al., 2023) and 6.5-22.9% of hyponatremia can be attributed to CSW in non-traumatic SAH patients (Sherlock et al., 2006; Kao et al., 2009; Hoffman et al., 2018). Older studies observed mild hyponatremia (Na 130-134 mmol/L) in up to 57% of non-traumatic SAH patients within the first week post-ictus (Yee et al., 2010).

CSW was first described in 1950 in a case report of 3 patients who developed hyponatremia (Peters et al., 1950). Hyponatremia following cerebral disease was predominantly attributed to the syndrome of inappropriate antidiuretic hormone secretion (SIADH) (Yee et al., 2010), as published by Schwartz et al. (1957) in 1957. In 1981, Nelson et al. (1981) questioned the diagnosis of SIADH in 12 patients, using the term CSW. SIADH is characterized by hyponatremia with mildly increased sodium excretion in the urine. Still, these patients present themselves as euvolemic to hypervolemic (Oh et al., 2015), while CSW can be distinguished primarily by hypovolemia and higher urine sodium excretion (Kaiya et al., 2019). It is essential to understand CSW symptoms can also be met without CNS damage; therefore, some authors prefer the term “renal salt wasting” (RSW) (Maesaka et al., 2009).

Although the criteria for diagnosing CSW are controversial, most authors agree that it causes excessive natriuresis, leading to hyponatremia and hypovolemia. It is essential to exclude renal impairment, heart failure, thyroid impairment, and common causes of pseudohyponatremia when assessing suspected CSW (Oh et al., 2015).

While the exact etiology of CSW remains contested, impaired sympathetic nervous system function and changes in serum natriuretic peptides are amongst the most widely accepted (Yee et al., 2010; Oh et al., 2015).

**Fig. 1:**
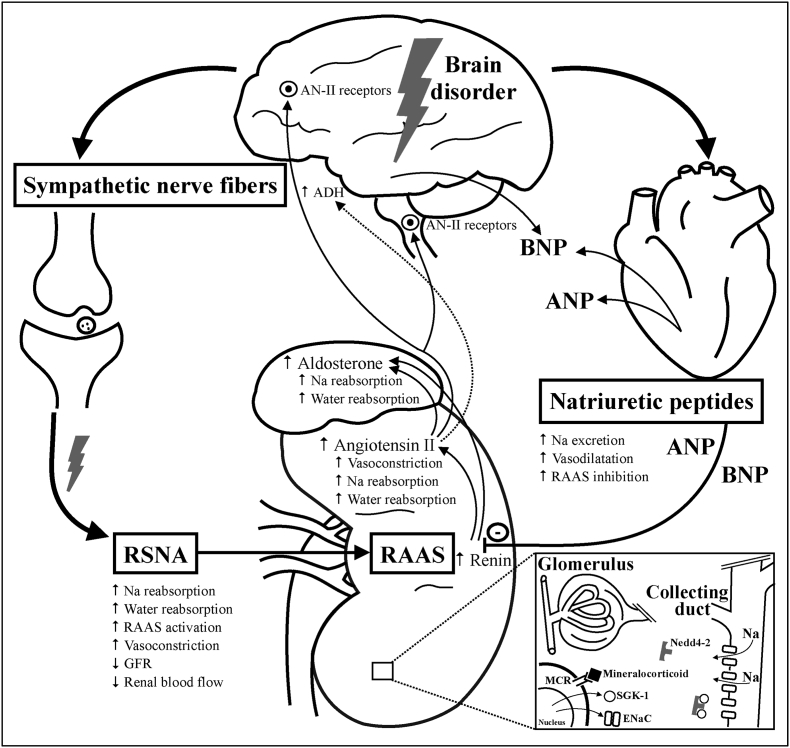
**Pathomechanism & mineralocorticoid effect** Abbreviations: RSNA^35^ = renal sympathetic nerve activity; RAAS = renin-angiotensin-aldosterone-system; Na = sodium; AN-II = Angiotensin II; GFR = glomerular filtration rate; ANP = atrial natriuretic peptide; BNP = brain natriuretic peptide; ADH = antidiuretic hormone; MCR = mineralocorticoid receptor; ENaC = epithelial sodium channels; SGK-1 = serum-and glucocorticoid-regulated kinase 1; Nedd4-2 = neural precursor cell expressed, developmentally down-regulated 4-Like, E3 ubiquitin protein ligase 2. Illustration of the pathomechanism for CSW according to the two most popular theories and the functional mechanism of mineralocorticoids in CSW patients. “Mineralocorticoids” represent corticosteroids with impact on mineralocorticoid receptors. Dexamethasone exhibits minimal mineralocorticoid effect, while hydrocortisone has significantly higher effect compared to dexamethasone.

Early studies showed that the use of synthetic corticosteroids with mineralocorticoid effects (Fig. 1), initially using fludrocortisone (Hasan et al., 1989; Mori et al., 1999) and later hydrocortisone (Moro et al., 2003; Katayama et al., 2007) (due to their shorter biological half-lives), was related to the prevention or at least reduction of natriuresis’ occurrence. Glucocorticoids, e.g., dexamethasone, have often been administered to non-traumatic SAH patients for their immunosuppressive and anti-inflammatory properties. This could reduce complications such as increased intracranial pressure due to brain swelling or delayed cerebral ischemia (DCI), improving outcomes (Feigin et al., 2005). Over time, many studies have shown that preventing CSW-associated hyponatremia using corticosteroids has become more critical (Treggiari et al., 2023; Busl and Rabinstein, 2023). A cross-sectional survey from 2011 showed that 57% of high-volume centers in the US reported routine dexamethasone administration after SAH, although the intended purpose varied (Tomycz et al., 2011). There have been only three randomized controlled trials (RCT) (Hasan et al., 1989; Mori et al., 1999; Katayama et al., 2007) on using mineralocorticoids for hyponatremia in SAH patients, with only one investigating hydrocortisone (Katayama et al., 2007). The study showed that hydrocortisone helped prevent excess natriuresis and hyponatremia (Katayama et al., 2007). Over time, a change in standard prophylactic corticosteroid treatment for non-traumatic SAH patients occurred in our department, transitioning from dexamethasone to hydrocortisone. This enabled a retrospective comparison of the two treatment regimens.

The aim of this study was to compare the incidence of hyponatremia and CSW in non-traumatic SAH patients receiving prophylactic corticosteroid treatment during neurocritical care.

## 2 Methods

### 2.1 Study design

We present a retrospective comparison of dexamethasone versus hydrocortisone in a historical cohort of patients with SAH.

A total of 510 patients were enrolled in this study. We performed a retrospective analysis of the sodium levels during dexamethasone *(n=*188) and hydrocortisone *(n=*322) administration in non-traumatic SAH patients treated in our department from October 2009 to December 2019. Except for individual cases, our clinic's internal treatment regimen consisted of prophylactic dexamethasone (4 mg TID for 7 days) until March 2014. It was then switched to hydrocortisone (200 mg per day as continuous infusion for 4 days, followed by 100 mg per day on the fifth day, and subsequent tapering over the next two days). All consecutive non-traumatic SAH patients who received either of the two corticosteroids within the defined timeframe were included. The aim was to compare hyponatremia (<130 mmol/l), the incidence of CSW (negative balance and hyponatremia <135 mmol/l), as well as the influence of the corticoid dose on both conditions between the groups. The threshold of sodium <130 mmol/l was chosen to identify hyponatremia with increased clinial und therapeutic relevance, whereas mild hyponatremia (<135 mmol/l) occurs more frequently and is often transient in SAH patients.

Blood sodium levels were monitored multiple times per day. Daily fluid management aimed at achieving a balanced net fluid status (±mL/24h). Patients diagnosed with “CSW” received fludrocortisone as treatment. In these cases, post-fludrocortisone sodium levels were excluded from further analysis.

Informed consent was waived due to the retrospective design of this study, with inclusion dates starting from 2009. All methods were carried out in accordance with relevant guidelines and regulations, and protocols were approved by the ethics committee of the University of Münster (2023-509-f-S).

### 2.2 Data collection

Data from all non-traumatic SAH patients were extracted from the electronic medical record. Patients were included if corticosteroid administration was commenced within 96 h post-ictus (Table 2). Fifty-eight *(n=*58) patients who received both corticosteroid drugs during their hospitalization were assigned to the medication they received first.

Patients who received corticosteroids before admission, or prophylactic corticosteroid treatment for fewer than 3 days during admission, were excluded. A total of 25 patients were excluded from statistical analysis.

Of 510 patients, 38 received additional fludrocortisone treatment for CSW during admission. For these patients, only data collected prior to fludrocortisone administration were considered.

Plasma sodium baseline levels were recorded upon admission, and further sodium levels were documented during the prophylaxis period. Only plasma sodium measurements obtained on days with active corticosteroid administration were included in the statistical analyses. Follow-up for sodium measurements was censored at the time of corticosteroid discontinuation, and no sodium values obtained thereafter were included in the analysis.

The minimum plasma sodium concentration per patient-day was compared between both groups. Due to the lack of comprehensive measurements of urine osmolality and sodium in our cohort, the criteria for CSW diagnosis were defined as plasma sodium levels <135 mmol/L and a simultaneous negative fluid balance on the same day. The latter was chosen precisely based on previous publications, all of which struggled to define fluid status or hypovolemia, as no standardized diagnostic approach has been established yet (Table 1).

**Table 1:**
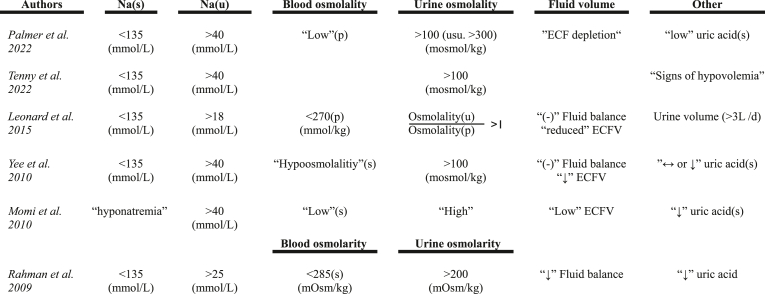
Diagnostic criteria for CSW.

List of possible diagnostic criteria for CSW, according to different publications.

Arrow symbols (↓/↔), the notation “(−)” and terms enclosed in quotation marks ('') used in this table reflect their usage in the referenced studies and were not further explained in these sources.

(s) = serum; (p) = plasma; (u) = urin; (L/d) = liters per day; ECF = extracellular fluid; ECFV = extracellular fluid volume.

Adapted from:

UpToDate. Cerebral salt wasting. 2023. https://www.uptodate.com/contents/cerebral-salt-wasting (accessed Sep 9, 2023).

StatPearls. Cerebral Salt Wasting Syndrome. 2022. https://www.ncbi.nlm.nih.gov/books/NBK534855/(accessed Aug 2, 2023).

Leonard J, Garrett RE, Salottolo K, et al. Cerebral salt wasting after traumatic brain injury: a review of the literature. Scand J Trauma Resusc Emerg Med. 2015; 23(1). https://doi.org/10.1186/S13049-015-0180-5.

Yee AH, Burns JD, Wijdicks EFM. Cerebral salt wasting: pathophysiology, diagnosis, and treatment. Neurosurg Clin N Am. 2010; 21(2):339-352. https://doi.org/10.1016/J.NEC.2009.10.011.

Momi J, Tang CM, Abcar AC, Kujubu DA, Sim JJ. Hyponatremia—What Is Cerebral Salt Wasting? Perm J. 2010; 14(2):62. https://doi.org/10.7812/TPP/08-066.

Rahman M, Friedman WA. Hyponatremia in neurosurgical patients: clinical guidelines development. Neurosurgery. 2009; 65(5):925-936. https://doi.org/10.1227/01.NEU.0000358954.62182.B3.

**Table 2:** Characteristics of the study population.

	Dexamethasone	Hydrocortisone	*p*
	(n = 188)	(n = 322)	
Sex			*0.1590*
Male, n (%)	66 (35.11%)	134 (41.61%)	
Female, n (%)	122 (64.89%)	188 (58.39%)	
Age			*0.2503*
Median in years (range) (IQR)	55 (28-88) (47.75-64.50)	57 (22-94) (49-66)	
BMI			*0.1325*
Median (range) (IQR)	25 (17.30-42.20) (23.38-27.10)	25.50 (17.70-42.80) (23.40-27.88)	
Fisher Score			*0.2012*
I, n (%)	2 (1.06%)	13 (4.04%)	
II, n (%)	12 (6.38%)	15 (4.66%)	
III, n (%)	64 (34.04%)	131 (40.68%)	
IV, n (%)	104 (55.32%)	156 (48.45%)	
Missing, n (%)	6 (3.19%)	7 (2.17%)	
Angio negative SAH, n (%)	43 (22.87%)	98 (30.43%)	*0.0808*
Aneurysma location			*0.1309*
ICA (siphon or bifurcation), n (%)	8 (4.26%)	25 (7.76%)	
MCA, n (%)	39 (20.74%)	47 (14.60%)	
AcoA, n (%)	50 (26.60%)	88 (27.33%)	
PcoA, n (%)	19 (10.11%)	28 (8.70%)	
Basilar artery, n (%)	14 (7.45%)	15 (4.66%)	
Vertebral artery, n (%)	5 (2.66%)	11 (3.42%)	
Other (unspecified) arteries, n (%)	27 (14.36%)	39 (12.11%)	
Other (unspecified) SAB, n (%)	26 (13.83%)	69 (21.43%)	
Intervention			
Clipping, n (%)	63 (33.51%)	70 (21.74%)	*0.0047*
Coiling, n (%)	88 (46.81%)	151 (46.89%)	*1.0000*
Decompression, n (%)	30 (15.96%)	37 (11.49%)	*0.1742*
Arterial spasmolysis, n (%)	52 (27.66%)	57 (17.70%)	*0.0099*
Baseline sodium (p)			*0.1833*
Median in mmol/L (range) (IQR)	137 (123-155) (135-139)	138 (123-149) (136-140)	
Median cortisone onset after admission in days (range) (IQR)^a^	1 (0-4) (0-1)	0 (0-4) (0-1)	
Median cortisone application in days (range) (IQR)	9 (1-37) (5-15)	10 (1-42) (8-12.75)	
Mean cortisone application per day in mg (±SD)	9.21 (4.32)	114.30 (82.00)	
Eq. dose^b^	229.27 (108.69)		
Median cortisone application per day in mg (IQR)	12 (6-12)	100.80 (35-201.60)	
Eq. dose^b^	300 (150-300)		

n = number of patients; (p) = plasma; ICA = internal carotid artery; MCA = middle cerebral artery; AcoA = anterior communicating artery; PcoA = posterior communicating artery;

(IQR) = interquartile range; (±SD) = standard deviation.

a Note that the first hospital day for each patient was only considered for establishing baseline sodium levels, and corticosteroid doses administered on the admission day were not included in the statistical analysis.

b Equivalent dose of dexamethasone: actual dose multiplied by 25.

The duration of corticosteroid prophylaxis (in days) and the equivalent hydrocortisone dose (in mg) were compared between the two groups. Daily equivalent dose conversion was performed for 1 mg dexamethasone, equal to 25 mg hydrocortisone, due to their different glucocorticoid and mineralocorticoid effects (Table 3). Baseline serum sodium concentration, patient age, daily corticosteroid dose (calculated as hydrocortisone equivalent), BMI, sex, aneurysm treatment modality (clipping vs. coiling), and decompressive craniectomy were considered potential confounders in the statistical analyses.

**Table 3:** Potency of systemic steroids.

	Glucocorticoid activity (relative)	Mineralcorticoid activity (relative)	Equivalent dose in mg
Hydrocortisone	1	1	20
Dexamethasone	25-30	0	0.75
Fludrocortisone	10	250	

Comparisons of the potency of different synthetic steroids.

mg = milligram.

Adapted from:

Adcock IM, Mumby S. Glucocorticoids. In: Page CP, Barnes PJ, editors. Pharmacology and Therapeutics of Asthma and COPD. Cham: Springer International Publishing, 2016. p. 171–196.

Asare K. Diagnosis and treatment of adrenal insufficiency in the critically ill patient. Pharmacotherapy. 2007; 27(11):1512-1528. https://doi.org/10.1592/PHCO.November 27, 1512.

Paragliola RM, Papi G, Pontecorvi A, Corsello SM. Treatment with synthetic glucocorticoids and the hypothalamus-pituitary-adrenal axis. Int J Mol Sci. 2017; 18(10). https://doi.org/10.3390/IJMS18102201.

### 2.3 Statistical analysis

Statistical analyses were performed using the R-Studio software (Version 4.3.2). Poisson and Negative Binomial models were used to represent the relationship between the number of events, Na(p) < 130 per patient, and the explanatory variables considered. Our study was based on findings from the first three weeks after ictus; therefore, no further follow-up data were extracted from the electronic medical records.

We chose a negative binomial model over the Akaike information criterion (AIC) due to the overdispersion of the count data. Residual checks and zero-inflation tests were carried out for the model. We used a log link function to better model the mean of the counted data as a linear combination of the explanatory variables, allowing for a comparison of hyponatremia events between the two groups. The same exposure variables were applied to a Generalized Linear Mixed Model (GLMM) with a random slope for Bernoulli trials (Binomial family, logit link) and optimization by quadratic approximation (BOBYQA) to model events with Na(p) < 130 as the binary dependent variable. The random effects model here enables us to consider the dependence of the observations due to the repeated measures design across time for each patient.

We performed a Wald test, defining *p*-values <0.05 as significant.

Univariate baseline comparisons for Table 2 were performed using the Mann-Whitney-U-test for continuous variables and Fisher's exact test for categorical variables with two categories. Variables with more than two categories were analyzed using Pearson's chi-square test.

**Fig. 2:**
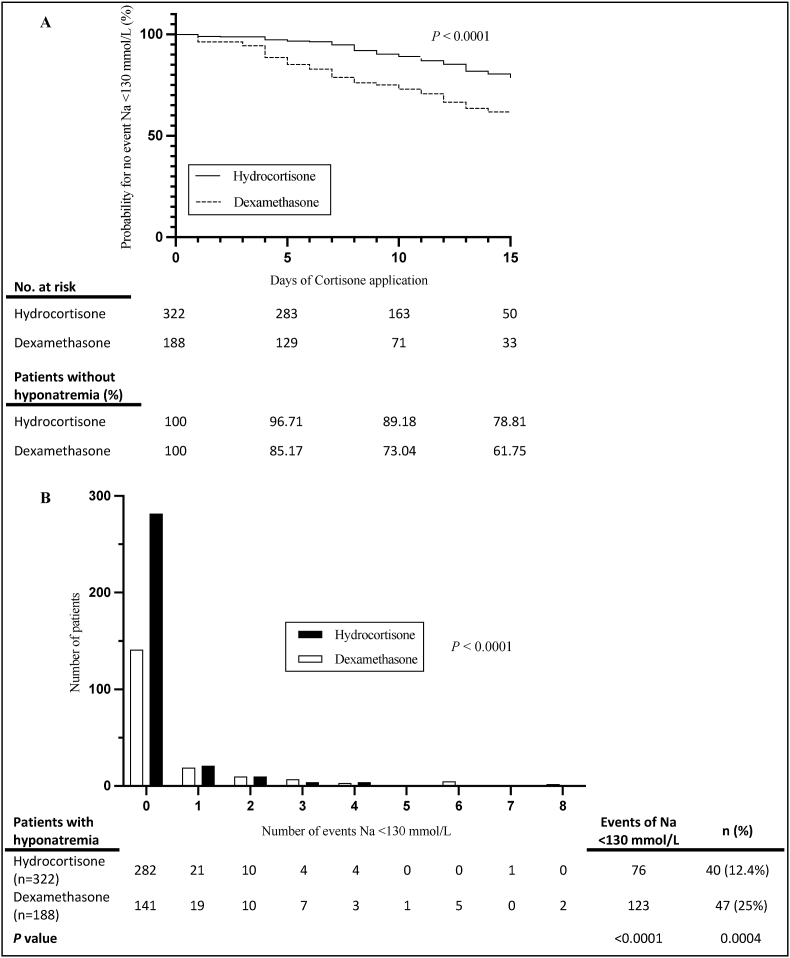
**Hyponatremia occurrence and distribution (A)** Kaplan Meier curve for the occurrence of events Na(p) < 130 mmol/L during prophylactic application of Hydrocortisone and Dexamethasone. **(B)** Bar graph illustrating the distribution of events Na(p) < 130 mmol/L between Hydrocortisone and Dexamethasone, highlighting variances during prophylactic corticosteroid administration. Abbreviations: Na = sodium; (p) = plasma; No = number; *n =* number of patients.

Separately, an exact Fisher test (Fig. 2), a log-rank test for creating a Kaplan Meier curve (Fig. 2), and a Chi-square test for making a bar graph with event distribution (Fig. 2), as well as for assessing CSW occurrence, were carried out using the Graph Pad Prism 10 software. For sensitivity analyses using a stricter CSW definition (hyponatremia <130 mmol/L combined with negative fluid balance), Fisher's exact test was used.

## 3 Results

Baseline characteristics were comparable between both groups, with significant differences observed only in the rates of arterial spasmolysis and aneurysm clipping (Table 2).

### 3.1 Corticosteroid administration

Prophylactic dexamethasone was administered for a median of 9 days (range: 1-37) and hydrocortisone for 10 days (range: 1-42), with mean daily doses of 9.21 ± 4.32 mg of dexamethasone per day and 114.27 ± 81.88 mg of hydrocortisone per day (Table 2). Considering that hydrocortisone, in contrast to dexamethasone, exhibits markedly stronger mineralocorticoid activity, this corresponds to an equivalent dexamethasone dose of 229.27 ± 108.69 mg, calculated as the administered dexamethasone dose multiplied by 25.

### 3.2 Incidence and severity of hyponatremia

Two hundred forty (47.1%) patients (regardless of their prophylaxis) showed hyponatremia (Na(p) < 135 mmol/L) within the first seven days following admission. One hundred and two patients (20%) showed sodium levels <135 mmol/L on the day of admission. Hyponatremia (Na(p) < 125 mmol/L) occurred in eight (4.3%) dexamethasone patients and ten (3.1%) hydrocortisone patients. One patient in each group showed sodium levels <125 mmol/L between admission and the start of corticosteroid treatment.

### 3.3 Diagnosis of CSW

Of 510 patients, 203 (39.80%) (89 dexamethasone (47.34%) vs. 114 hydrocortisone patients (35.40%)) met our predefined criteria for CSW by showing simultaneous hyponatremia (Na(p) < 135 mmol/L) as well as negative fluid balance *(p* = 0.0079, OR: 1.64, 95% CI: 1.14-2.37) during prophylactic corticosteroid as an expression of CSW depending on the chosen diagnostic criteria (Table 1). Using a stricter CSW definition (hyponatremia <130 mmol/L combined with negative fluid balance) 62 of 510 patients (12.16%) (33 dexamethasone (21.29%) vs. 29 hydrocortisone patients (9.90%) met the criteria (*p* = 0.007, OR 2.15, 95% CI: 1.26-3.67).

This corresponded to a significantly higher risk of CSW in the dexamethasone group.

### 3.4 Development and risk factors for hyponatremia

The groups showed significant differences in the development of hyponatremia (Na(p) < 130 mmol/L) measured over the elapsed time since the start of prophylactic corticosteroid treatment (Fig. 2). Kaplan–Meier analysis demonstrated that after seven days of corticosteroid administration, approximately 95% of hydrocortisone-treated patients and 79% of dexamethasone-treated patients remained free from hyponatremia, indicating a significantly lower incidence under hydrocortisone treatment (*p* < 0.0001, Fig. 2).

Multiple episodes of hyponatremia (>1 event) occurred more frequently among dexamethasone-treated patients (28 of 188 patients, 14.9%) than among those receiving hydrocortisone (19 of 322 patients, 5.9%) (*p* < 0.0001), suggesting that hyponatremia tended to occur more often and/or persist longer under dexamethasone prophylaxis.

Seven patients from the dexamethasone group and three from the hydrocortisone group showed sodium levels <130 mmol/L within the first corticosteroid administration day. In the dexamethasone group, 5 of 7 patients had a baseline sodium level of <135 mmol/L, and 3 of 7 had a level of <130 mmol/L. In the hydrocortisone group, two of three patients had baseline sodium levels <135 mmol/L, and only one showed levels <130 mmol/L. Higher baseline sodium levels were linked to a lower risk of developing hyponatremia during hospitalization (OR = 0.86, 95% CI: 0.84-1.036). A decrease of 5 mmol/L in baseline sodium increased the odds of developing hyponatremia by approximately 2.1-fold (OR = 2.14).

In contrast, increasing age was associated with a higher likelihood of developing hyponatremia, with the odds rising by approximately 3.4% per year of age, corresponding to about a 39% increase over ten years (OR = 1.034, 95% CI: 1.003-1.066, *p* = 0.032), indicating that advancing age is a significant risk factor for hyponatremia.

Although this association did not reach statistical significance, higher corticosteroid doses (calculated as hydrocortisone equivalents) were associated with a numerically lower odds developing hyponatremia (OR = 0.997, 95% CI: 0.993-1.000).

None of the further potential confounders included in the statistical analysis showed a statistically significant association with the occurrence of hyponatremia.

### 3.5 Comparative analysis: Dexamethasone vs. Hydrocortisone

**Fig. 3:**
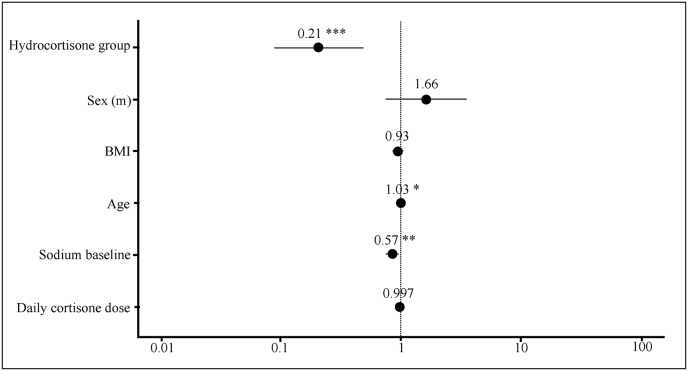
**Odds for hyponatremia**. Forest plot illustrating the odds ratios (OR) and 95% confidence intervals (CI) for the association of various factors with events of hyponatremia <130 mmol/L. Abbreviations: (m) = male; BMI = Body Mass Index; (∗∗∗) = *P* < 0.001; (∗∗) = *P* < 0.01; (∗) = *P* < 0.05.

In adjusted regression analysis, the data showed a significant difference between the two groups and an increased risk of developing hyponatremia (Na(p) < 130 mmol/L) (OR = 0.207, 95% CI: 0.090-0.477, *p* = 0.00021) (Fig. 3). Prophylactic corticosteroid use with hydrocortisone appeared to be protective against events of Na(p) < 130 mmol/L, and the estimated odds for a plasma sodium level <130 mmol/L are 4.8 times higher for patients who received dexamethasone (1/0.207 = 4.8). As shown in Figs. 2 and 25% of dexamethasone-treated patients and 12.4% of the hydrocortisone group developed hyponatremia (Na(p) < 130 mmol/L), despite a 1:2 patient ratio (dexamethasone vs. hydrocortisone) and an overall higher number of events for hyponatremia <130 mmol/L (*p* < 0.0001). These proportions represent unadjusted event rates and are therefore not directly comparable to the adjusted effect estimates derived from regression modeling.

## 4 Discussion

To date, this is the largest retrospective study demonstrating that hydrocortisone is associated with a lower risk of developing hyponatremia and CSW compared to dexamethasone. In this study, we compare the prior local standard of care, dexamethasone, with hydrocortisone in SAH patients, considering that hydrocortisone, unlike dexamethasone, has a mineralocorticoid effect. We postulate that prophylactic hydrocortisone administration is more effective in preventing CSW-associated hyponatremia following non-traumatic SAH.

Hydrocortisone prophylaxis could significantly reduce the occurrence of hyponatremia (<130 mmol/L) *(p<*0.001), while hyponatremia was 4.8 times more likely in the dexamethasone group.

### 4.1 Hyponatremia after aSAH: implications and controversies

CSW leads to extreme natriuresis with simultaneous hypovolemia (Yee et al., 2010). Hyponatremia is associated with a more extended hospital stay and an increased risk for complications such as vasospasm, seizures, or cerebral ischemia (Rahman and Friedman, 2009; Mapa et al., 2016). Currently, there is no consensus on whether hyponatremia increases the risk of mortality (Kaiya et al., 2019; Rahman and Friedman, 2009) or does not affect it (Sherlock et al., 2006; Mapa et al., 2016).

A similar discussion applies to the risk of hyponatremia, which appears to be unrelated to the severity of SAH, as measured by the Hunt and Hess grading scale, in some studies (Marupudi and Mittal, 2015), whereas others have reported a significant correlation (Kieninger et al., 2021; Hannon et al., 2014a). However, the risk of a CSW itself seems to correlate with the severity of the bleeding as indicated by the Hunt and Hess grading scale (Hoffman et al., 2018). The Fisher score was normally distributed across both treatment groups, with no statistically significant difference between them (*p* = 0.2012). Therefore, it was not included in the multivariate statistical models used for the primary analysis.

While earlier studies found no association between aneurysm location and the occurrence of hyponatremia, more recent data indicate that anterior circulation aneurysms may predispose to hyponatremia, most commonly due to SIADH, likely reflecting the proximity of these aneurysms to the hypothalamic–pituitary axis (Hannon et al., 2014b). As aneurysm location was comparable between treatment groups (*p* = *0.1309*), it was not included in the multivariate analysis.

In previous studies, no significant association has been demonstrated between aneurysm treatment - surgical clipping or endovascular coiling - and the occurrence of hyponatremia. The impact of clipping remains uncertain, as some studies (Hannon et al., 2014a) found no correlation, whereas others did (Sherlock et al., 2006). In our cohort, no significant difference was observed between clipping and coiling regarding the development of hyponatremia (*p* = 0.402), supporting previous findings that the treatment method does not appear to influence the risk of hyponatremia.

Similarly, our data showed no significant association between decompressive craniectomy (*p* = 0.3067) and the development of hyponatremia.

### 4.2 Challenges in CSW diagnosis: varying volume depletion

CSW diagnosis and its differentiation from SIADH remains challenging (Maesaka et al., 1999) due to the various proposed diagnostic approaches (Table 1) and, most importantly, the lack of consistent methods for evidence of volume depletion in CSW patients (Sterns and Silver, 2008). The most crucial parameter to differentiate CSW from SIADH is hypovolemia (Oh et al., 2015).

There is no established standard for determining volume depletion despite the use of various methods in previous studies. These methods have included clinical signs of hypovolemia such as decreased blood pressure, central venous pressure, and weight loss, or the use of isotope dilution and hematocrit measure (Verbalis, 2020). This might explain the varying severity and frequency of CSW diagnoses in different publications (Verbalis, 2020). Some authors suggest monitoring fluid balance (Yee et al., 2010), while others use blood and urine osmolality to define fluid status (Maesaka et al., 2007). Assessment of volume depletion is further complicated by the fact that extracellular fluid does not necessarily correlate with intravascular fluid in critically ill patients.

We used daily fluid balances to define volume depletion since other methods of blood volume assessment had only been documented for a subset of patients. The same applies to criteria like plasma or urine osmolality and sodium excretion in urine (Katayama et al., 2007).

### 4.3 Pathomechanism theories

Both popular theories for CSW (Fig. 1) have yet to be proven valid, with a combination of both pathomechanisms also conceivable (Dong and Kwon, 2009).

Brain damage may disrupt the sympathetic influence on the kidney, according to the “sympathetic theory”. Besides the RAAS (renin-angiotensin-aldosterone system), the kidney's salt- and water regulation is influenced via sympathetic nerve fibers, which are connected to the tubule, the vessels of the kidney, and the juxtaglomerular cells (Johns et al., 2011). DiBona (2000) calls this connection “RSNA” (renal sympathetic nerve activity). Typically, a volume reduction triggers increased RSNA activity in the kidney, leading to more significant water and sodium reabsorption, vasoconstriction-induced decrease in glomerular filtration rate (GFR), and RAAS activation (Fig. 1). (Yee et al., 2010) Initially, renin increases aldosterone and angiotensin II (AN-II) release (DiBona, 2000). Aldosterone affects kidney tubules, causing water and sodium reabsorption. AN-II acts directly within the central nervous system and influences the sympathetic nervous system through binding sites in the forebrain and brainstem, playing an essential role in RSNA regulation (DiBona, 2000).

The result of an RSNA disorder would be sodium and water loss and reduced synthesis of renin and aldosterone, as seen in patients with SAH and CSW patients (Momi et al., 2010).

Two case reports of RSW patients showed increased aldosterone and renin (Maesaka et al., 2007; Bitew et al., 2009). In addition, experiments with induced SAH in dogs indicate an increased sympathetic nervous tone after such brain damage (Jacob et al., 1972). This theory suggests no increase in AT-II with reduced RAAS activity in the kidney. However, this was precisely demonstrated in a study with SAH in rat models (Takemoto et al., 2020), despite the study not specifically examining the connection between hyponatremia and CSW.

There's another theory for CSW development, known as the “natriuretic peptide theory.” Natriuretic peptides, primarily released by cardiomyocytes, regulate water and electrolyte balance in the body (Goetze et al., 2020). They increase diuresis via sodium excretion, vasodilation, and inhibition of RAAS and the influence of the sympathetic nervous system (Fig. 1). (Weber and Hamm, 2006) Increased brain natriuretic peptide (BNP) could be observed in SAH patients with hyponatremia, allegedly due to CSW (Berendes et al., 1997). Others consider that BNP plays a more decisive role in pathophysiology than, e.g., atrial natriuretic peptide (ANP) (Yee et al., 2010; Kao et al., 2009). BNP could also be detected in different brain areas, like the hypothalamus (Takahashi et al., 1992). In case of brain damage, a BNP release is possible as a stress reaction of the body to prevent an increase in intracranial pressure (Berendes et al., 1997). BNP induces diuresis and directly correlates with the risk of hyponatremia and vasospasms in SAH patients (McGirt et al., 2004). However, one study found no association between hyponatremia and BNP concentration in rat models exhibiting CSW after SAH (Kojima et al., 2005).

### 4.4 The prophylactic use of synthetic corticosteroids

Adrenal cortex produces three corticosteroids: glucocorticoids that affect metabolism and immune system, mineral corticosteroids that regulate salt and water balance, and androgens (Lorraine et al., 2003). Aldosterone is the main mineralocorticoid that interacts with the mineralocorticoid receptor (MCR) (Rudolph, 2004). When bound to the MCR, aldosterone induces it to act as a transcription factor for various genes, including those encoding serum- and glucocorticoid-regulated kinase-1 (SGK-1), leading to increased expression of epithelial sodium channels (ENaC) and sodium-potassium ATPase (Fuller and Young, 2005). SGK-1 phosphorylates the ubiquitin ligase “Nedd4-2" (neural precursor cell expressed, developmentally down-regulated 4-Like, E3 ubiquitin protein ligase 2), which can no longer mark ENaC for proteasomal degradation afterward, increasing the number of ENaC in the apical nephron membrane (Viengchareun et al., 2007) (Fig. 1). MCR stimulation leads to increased sodium reabsorption in the kidney, resulting in osmotic water absorption and increased secretion of potassium and hydrogen (Norman et al., 2014a). Unlike glucocorticoid receptors (GCR), MCRs can interact with aldosterone and cortisol as ligands, but aldosterone binding is more stable (Norman et al., 2014b). It is known that MCRs have a high affinity for glucocorticoids and, therefore, already react to low glucocorticoid concentrations (Scherholz et al., 2019). To prevent overstimulation of the MCR by glucocorticoids, the 11β-hydroxysteroid dehydrogenase 2 (11B-HSD2) in epithelial tissues (such as the kidney) converts cortisol into its less active form, cortisone (Fuller et al., 2012).

Synthetic corticosteroids, such as hydrocortisone, dexamethasone, or fludrocortisone, can also stimulate the MCR or GCR (Fig. 1). Still, their mineralocorticoid and glucocorticoid potencies vary due to differences in chemical structure and modifications (Adcock and Mumby, 2016; Samuel et al., 2017) (Table 3). Dexamethasone and hydrocortisone are often classified as “glucocorticoids” with mineralocorticoid potencies, while fludrocortisone is classified as a “mineralocorticoid” because of its high mineralocorticoid and minimal glucocorticoid activity (Samuel et al., 2017). Administering hydrocortisone prophylactically could prevent excessive natriuresis as it has higher mineralocorticoid potency than dexamethasone (Table 3).

Two studies (Moro et al., 2003; Katayama et al., 2007), analyzed the prophylactic effect of hydrocortisone in SAH patients who received 1200 mg/d hydrocortisone for ten days. Their results showed that hyponatremia could be prevented. Patients' hypovolemia seemed affected despite hypervolemic fluid administration. Hydrocortisone benefits in countering sodium excretion after SAH were confirmed in a rat model (Yoneko et al., 2010). Hydrocortisone usually does not affect the MCR due to prevention by 11B-HSD2, and events such as hypoxia could limit 11B-HSD2 activity.

A review (Shah et al., 2018) found that fludrocortisone and hydrocortisone can limit natriuresis, but RCTs evaluating their effect on sodium showed selection bias in reporting standard functional outcomes (Hasan et al., 1989; Mori et al., 1999; Katayama et al., 2007). However, they agreed on the benefits of using mineralocorticoids in limiting natriuresis. Although fludrocortisone has been proven to be effective against hyponatremia and is often administered in CSW patients, current guidelines only recommend mineralocorticoid treatment to a limited extent [Class of recommendation (COR): 2a, Level of Evidence (LOE): B-R] (Hoh et al., 2023). Furthermore, the latest guidelines from the Neurocritical Care Society recommend using mineralocorticoids to manage hyponatremia after aSAH (Treggiari et al., 2023). It is based solely on four publications (Hasan et al., 1989; Mori et al., 1999; Moro et al., 2003; Katayama et al., 2007). Only 1 RCT (Katayama et al., 2007) investigates the effect of hydrocortisone for hyponatremia prophylaxis. All four studies share “a high risk of bias and imprecision due to small sample sizes and methodological limitations” (Treggiari et al., 2023). Therefore, the authors suggested more extensive trials (Treggiari et al., 2023).

Our retrospective study's generalizability is strengthened by the analysis of the largest cohort of non-traumatic SAH patients *(n=*510) published over a decade, which investigated the prophylactic administration of synthetic corticosteroids for preventing hyponatremia and provided valuable insights into the effectiveness of corticosteroid prophylaxis at a large academic institution.

In conclusion, hydrocortisone in non-traumatic SAH patients seems effective in preventing hyponatremia. Definitive confirmation, however, requires prospective, multicenter studies.

Despite positive results for hyponatremia prevention, there is currently no recommendation for prophylactic glucocorticoid administration in SAH patients – partly due to reported adverse effects such as increased blood glucose and gastrointestinal bleeding (Feigin et al., 2005). Furthermore, a significant association between post-SAH treatment with dexamethasone and increased rates of systemic and EVD-related infections has been observed (Czorlich et al., 2017). To date, no study has shown a worsening of hyponatremia/CSW due to corticosteroid administration (Feigin et al., 2005; Shah et al., 2018).

The use of mineralocorticosteroids in patients with SAH for the prevention or treatment of hyponatremia remains controversial. Differentiation between SIADH and cerebral salt wasting remains challenging in clinical practice, which may affect therapeutic decisions regarding the use of mineralocorticosteroids. While administration in combination with volume replacement in cases with a strong clinical suspicion of CSW is reasonable, prophylactic use remains controversial and should be interpreted cautiously in the context of this study.

Several potential confounders should be acknowledged. First, individual treatments during admission for electrolyte and fluid balance, such as saline infusion or the use of diuretics, may have affected the analyzed parameters. Additionally, the lack of standardized electrolyte management across all patients may have further contributed to variability in the results.

Second, pre-existing conditions such as kidney failure or cardiovascular disease, and consequently the intake of medications like diuretics, antidepressants, and antiepileptics, among others, could have influenced the development of volume status and sodium balance.

Due to predefined criteria for statistical analysis, additional potential confounders not included in this study - such as aneurysm location, SAH severity (Fisher or Hunt & Hess scores) or adjunctive treatments such as spasmolysis - must be considered.

### 4.5 Recommendation

Future research should focus on developing a uniform definition of CSW. Subsequently, the preventive effects of hydrocortisone on CSW (as defined uniformly) should be assessed in a prospective randomized trial.

### 4.6 Limitations

This study compares two standards of care for SAH in one department and their transition from 2009 to 2019. Its retrospective, single-center design limits how generalizable the findings are to other institutions or health care settings.

Although a 7-day course of prophylactic corticosteroids was intended, retrospective analysis showed longer median durations: 9 days for dexamethasone and 10 days for hydrocortisone. Evolving supportive care and treatment standards over the 10-year study period may have influenced outcomes. Non-standardized electrolyte management across patients may also have contributed to variability in the results.

Although corticosteroids have immunomodulatory effects, short-term, moderate-dose use, as in this study, has not consistently been linked to higher infection rates in SAH. However, infectious complications were not systematically assessed in this retrospective cohort and should be examined in future prospective studies.

Due to lack of a uniform definition of CSW, it is challenging to identify patients who have developed a CSW with hyponatremia. Additionally, the potentially necessary laboratory parameters for additional possible diagnostic criteria were not uniformly documented in our cohort. Urinary sodium excretion, urinary osmolality, and plasma osmolality - parameters some consider essential for a definitive, objective CSW diagnosis - were not routinely available in this retrospective cohort. Thus, CSW classification relied on hyponatremia (<135 mmol/L) and a negative daily fluid balance as a surrogate for possible hypovolemia. The 203 patients who met these criteria were identified during corticosteroid prophylaxis, so additional cases may have gone undetected.

## 5 Conclusion

Our findings suggest that hydrocortisone administration is associated with a lower incidence of CSW-associated hyponatremia development following non-traumatic SAH. According to our data from this retrospective, single-center study, hydrocortisone appeared more effective than dexamethasone in this context.

## Disclosures

None.

## Sources of funding

Open Access funding is provided by the Projekt DEAL.

## Declaration of interest

The authors declare that they have no known competing financial interests or personal relationships that could have appeared to influence the work reported in this paper.

## Data Availability

The data supporting this study's findings are available from the corresponding author upon request.
